# Aging in personal and social immunity: do immune traits senesce at the same rate?

**DOI:** 10.1002/ece3.1668

**Published:** 2015-09-18

**Authors:** Catherine E. Reavey, Neil D. Warnock, Amy P. Garbett, Sheena C. Cotter

**Affiliations:** ^1^ School of Biological SciencesQueen's University BelfastMBC97 Lisburn RoadBelfastBT9 7BLUK; ^2^ Lancaster Environment CentreLancaster UniversityLancasterLA1 4YQUK; ^3^ School of Life SciencesUniversity of LincolnBrayford PoolLincolnLN6 7TSUK

**Keywords:** Aging, defensin, ecological immunology, insect, lifespan, lysozyme, *Nicrophorus*, parental care, phenoloxidase, wounding

## Abstract

How much should an individual invest in immunity as it grows older? Immunity is costly and its value is likely to change across an organism's lifespan. A limited number of studies have focused on how personal immune investment changes with age in insects, but we do not know how social immunity, immune responses that protect kin, changes across lifespan, or how resources are divided between these two arms of the immune response. In this study, both personal and social immune functions are considered in the burying beetle, *Nicrophorus vespilloides*. We show that personal immune function declines (phenoloxidase levels) or is maintained (defensin expression) across lifespan in nonbreeding beetles but is maintained (phenoloxidase levels) or even upregulated (defensin expression) in breeding individuals. In contrast, social immunity increases in breeding burying beetles up to middle age, before decreasing in old age. Social immunity is not affected by a wounding challenge across lifespan, whereas personal immunity, through PO, is upregulated following wounding to a similar extent across lifespan. Personal immune function may be prioritized in younger individuals in order to ensure survival until reproductive maturity. If not breeding, this may then drop off in later life as state declines. As burying beetles are ephemeral breeders, breeding opportunities in later life may be rare. When allowed to breed, beetles may therefore invest heavily in “staying alive” in order to complete what could potentially be their final reproductive opportunity. As parental care is important for the survival and growth of offspring in this genus, staying alive to provide care behaviors will clearly have fitness payoffs. This study shows that all immune traits do not senesce at the same rate. In fact, the patterns observed depend upon the immune traits measured and the breeding status of the individual.

## Introduction

Senescence is the change in physiological processes and tissue function with age, exhibited in nearly all organisms (Stanley 2012). It results in a gradual loss of function at the level of the cells, tissue, and whole organism, culminating in some degree of irreversible decline with age. The rate of senescence shows a broad phylogenetic distribution. For example, *Drosophila melanogaster* live to 52 days, whereas Japanese women live to 102 years (5% of the population surviving) (Jones et al. 2013). Such a broad range of senescence rates across taxa indicates highly varied cellular and physiological processes, as well as widely different investment strategies.

Essentially, it is cumulative damage by biological processes that causes senescence (e.g., Kaszubowska 2008; Oliveira et al. 2010). Mechanisms such as antagonistic pleiotropy (Hughes and Reynolds 2005), adverse gene actions at older ages (Kirkwood and Austad 2000), and damage by reactive oxygen species (ROS) (Hoffmann 1995) can all contribute to senescence. Even with a very low rate of senescence, an organism will not live indefinitely due to environmental pressures. Therefore, the optimal investment strategy for all traits with respect to time will evolve in conjunction with externally imposed schedules of survival and reproduction (Kirkwood and Austad 2000); senescence is a key life‐history trait.

A central question in evolutionary biology is that of the proximate and ultimate reasons for changing investment patterns with age. The costs and benefits of a particular trait are likely to change across the lifespan of an individual. Investing in the immune system is one such trait that should vary substantially, either because of a decline in state and the changing risk of external mortality or because of changes in environment or behavior throughout the lifespan of a species (Rigby and Jokela 2000; Wilson et al. 2002; Lawniczk and Begun 2004). Furthermore, the value of investment in the immune system is likely to change as the individual ages. It is crucial that juvenile organisms maintain an efficient immune system in order that they reach adulthood and reproduce. If they do not, their direct fitness will be zero. We know that immune function is costly (Lenski 1988; Kraaijeveld and Godfray 1997; Koella and Boëte 2002; Hanssen et al. 2005; Lee et al. 2006; Sadd and Siva‐Jothy 2006; Valtonen et al. 2010; Simmons 2011), which raises the question of determining the optimal pattern of investment with age.

In insects, there are two main arms to the immune system: cellular immunity and humoral immunity. The standing immune response is the cellular system. A range of hemocytes act as the initial, generalized defense to invaders, using mechanisms such as phagocytosis of microparasites, nodulation of clumps of microparasites, and encapsulation of macroparasites (Gillespie et al. 1997). A main component of the constitutive response is the activation of the prophenoloxidase (pro‐PO) cascade (Gillespie et al. 1997). The end product of this cascade is melanin (Götz 1986), which is used for encapsulation. Further roles of phenoloxidase (PO) include involvement in non‐self‐recognition (Söderhäll and Cerenius 1998), coordination of the cellular response (Gillespie et al. 1997), and cuticular hardening (Sugumaran et al. 2000). Although present at a basal level, PO can be further activated and upregulated by a wide range of parasitic challenges (Gillespie et al. 1997). The PO cascade is also associated with humoral immunity; the intermediate quinones produced exhibit antimicrobial activity in the hemolymph (Nappi and Ottaviani 2000). Invasion also prompts a further induction of the humoral immune response, which is relatively specific in comparison with the generalized cellular response (Casteels et al. 1994; Lemaitre et al. 1997). The molecules involved include lysozyme and other small antimicrobial peptides (AMPs) (Hoffmann 1995). There are a huge range of AMPs, for example, cecropins, attacins, and defensins, to name a few (Hoffmann 1995). Defensin is a ubiquitous AMP found across the animal kingdom and forms part of the human immune response (Ganz 2003).

Various components of the personal immune system in insects have shown changes with age in the literature. PO has been observed to decrease with age in crickets, *Gryllus texensis* (Adamo et al. 2001), bumblebees, *Bombus terrestris* and *Bombus muscorum* (Moret and Schmid‐Hempel 2009; Whitehorn et al. 2011), and honeybees, *Apis mellifera* (Roberts and Hughes 2014). In contrast, in a study on the leaf‐cutting ant, *Acromyrmex octospinosus*, PO increases in older workers (Armitage and Boomsma 2010). Research by Li et al. (1992) showed a decrease in both PO associated with hemocytes and hemolymph PO with age. The encapsulation response has also been observed to decline in older age classes in *Bombus terrestris* (Doums et al. 2002). Older mosquitoes, *Aedes aegypti*, showed an age‐associated mortality in response to challenge and this corresponded to a decrease in hemocyte numbers (Hillyer et al. 2005). Hemocyte density has also been observed to decrease with age in *Bombus terrestris* (Moret and Schmid‐Hempel 2009). A decline in the nodulation response with age has been observed in several species; in honeybees, *Apis mellifera*, the number of nodules produced in response to freeze‐dried bacterial challenge declines with age (Bedick et al. 2001). In male crickets, *Gryllus assimilis*, declining numbers of nodules were formed in a graded response to lipopolysaccharide (LPS) injections with age (Park et al. 2011). Both hemocyte density and phagocytic ability have been shown to decline in *Drosophila melanogaster* (Mackenzie et al. 2011). *Drosophila* have yielded great insights into the genes involved in immunosenescence. Interestingly, the most obvious change in genome‐wide expression with age seems to be for the genes involved in the immune response (Sarup et al. 2011; Iliadi et al. 2012). Across studies, the increase in transcripts of immune response genes in *Drosophila melanogaster* with age has been observed (Pletcher et al. 2002; Zerofsky et al. 2005). These studies hypothesize that this upregulation may be due to a lifetime of exposure to pathogens, or it may result from a decline in the function of transcripts (Iliadi et al. 2012).

The above studies all consider changes in personal immune responses, but there are a suite of immune responses, called social immune responses (sensu Cotter and Kilner 2010b), that have been selected to increase the fitness of the challenged individual and one or more recipients (Cotter and Kilner 2010a). These responses occur across the animal kingdom. For example, the beewolf, *Philanthus triangulum*, provisions brood cells with bees upon which their offspring feed. The bee corpses are embalmed using a cocktail of hydrocarbons that create an environment unsuitable for fungal growth (Herzner and Strohm 2007; Herzner et al. 2007). Eggs of the three‐spined stickleback, *Gasterosteus aculeatus*, are protected from microbes by an antimicrobial mucus that glues the nest together (Little et al. 2008). Indeed, the provisioning of antibodies in mammalian milk also falls within the definition of a social immune response (Cotter and Kilner 2010a). Despite their ubiquity, we do not know how social immune responses change with age, or indeed how the balance of investment in personal and social immunity changes over an organism's lifetime.

**Figure 1:**
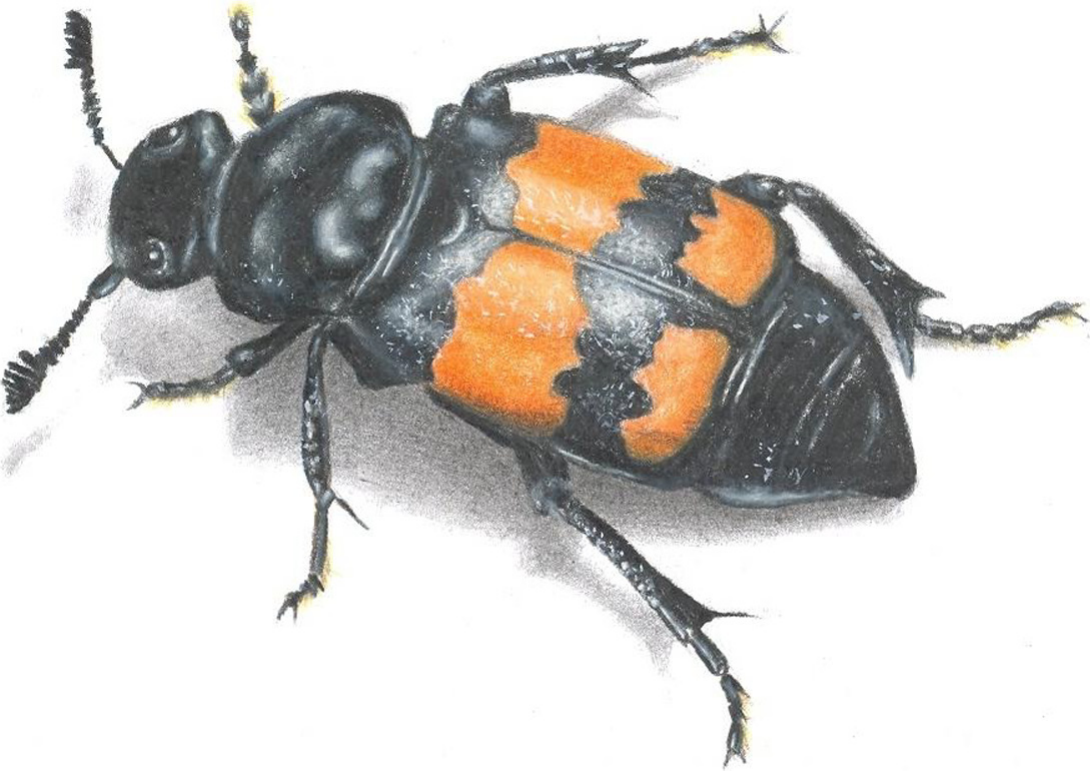
A *Nicrophorus vespilloides* female, courtesy of Steve Collett.

In this study, we look at patterns of immunity across lifespan in the burying beetle, *Nicrophorus vespilloides* (Fig. 1). This species is a carrion breeder and exhibits biparental care of young (Pukowski 1933). The parents cooperate to bury a small vertebrate carcass and prepare it for their offspring by removing hair or feathers and shaping it into a ball (Pukowski 1933; Scott 1998). Antimicrobial anal exudates are used to delay decomposition of the carcass (Cotter and Kilner 2010a), which is a form of social immunity (Cotter and Kilner 2010b). The antimicrobial exudates improve offspring survival; larvae do not develop as well on carcasses in an advanced state of decay (Rozen et al. 2008). Breeding success in this species drops off significantly as the beetles age (Cotter et al. 2010a). We know that the production of antimicrobial exudates, that is, social immunity, is costly (Cotter et al. 2010b) and that maintaining personal immunity is also costly (Reavey et al. 2014). Burying beetles therefore provide us with a system that allows us to easily consider both personal and social immune investment across lifespan. Is a change in the balance of these traits with age selected for?

We hypothesize that personal immune function will decline with age and that it will be suppressed in breeding beetles (Reavey et al. 2014), but perhaps this suppression will be exacerbated with old age when residual reproductive value declines. If social immunity follows a pattern of parental investment, we might expect an initial increase, but lower levels of lytic activity in later life. Currently, there are little or no studies on social immunity across lifespan or changes in the balance of personal and social immunity with age. Therefore, a central aim of this study was to further understand variation in personal and social immune function. Study of social immunity is very much in its early stages; the external social immune response is still part of the immune response but much less studied in terms of costs (Otti et al. 2014). Furthermore, many of the organisms in which social immunity has been studied are typically eusocial species in which the majority of individuals do not reproduce. Therefore, considering the balance of personal and social immunity in reproductive individuals is especially interesting, as both survival and reproduction are the central components contributing to fitness.

## Materials and Methods

### *Nicrophorus vespilloides*

The laboratory population was maintained as described previously (Reavey et al. 2014). In brief, non‐breeding adult beetles were housed in individual boxes containing moist soil at 20°C under a 16:8‐h light: dark cycle and fed twice weekly ad libitum with minced beef. Pairs were placed together in a breeding container, 1/3 filled with moist soil and provided with a mouse carcass. Containers were then placed in a dark cupboard to mimic underground conditions. Larvae were removed from the breeding container as soon as they began dispersing from the carcass, typically 8–10 days after the parents were paired, and placed individually in compartments of 25‐cell petri dishes, covered with moist soil and left to pupate. Eclosion occurs around 20 days following dispersal, after which the beetles were set up in their individual containers for either laboratory population beetles or for use in later experiments.

The mean lifespan of beetles from our pedigree data was 51.92 days ± 0.23, with mortality rising sharply thereafter. Adult age is measured from the point of eclosion, rather than since the hatching from the egg. Therefore, age classes for the experiments were selected from 0 to 8 weeks old. Discrete groups of beetles were used in each experimental setup. Different age classes were used across experiments; for non‐breeding beetles, PO was measured from 0 to 8 weeks. Due to the time required for reproductive maturation and a decline in breeding with age potentially providing less data, age classes from 2 to 7 weeks were used in experiments carried out on breeding beetles. When measuring antimicrobial activity (AMP – defensin), the age classes selected were 2, 5, and 8 weeks. This range was selected to cover as much of the lifespan of the beetles as possible in uniform intervals, but due to logistical constraints, more age classes could not be included.

### Experiment 1: Changes in personal and social immunity across lifespan

#### Changes in personal immunity (PO) across lifespan in breeding and nonbreeding beetles

Constitutive PO levels were measured in this part of the study. Firstly, PO activity in non‐breeding beetles across lifespan was measured. Standing levels of PO in non‐breeding beetles were measured on a weekly basis from 0 to 8 weeks of age, with week 0 being 2 days following eclosion. Hemolymph could only be sampled from each beetle once, as wounding alone will trigger an immune response (Reavey et al. 2014). Therefore, separate individuals in discrete groups (*n* = 18) were used for each age class (total sample size = 162 beetles). Due to death in the later stages of this experiment, some individuals did not provide samples. 130 samples were obtained in total. Individuals were fed mince ad libitum on the day prior to sampling, and sampling took place at the same time of day.

PO in breeding beetles across lifespan was then measured. Six age classes were used, beetles aged 2 weeks to 7 weeks at weekly intervals, with each age class consisting of a discrete group of female beetles. This experiment focused on females only in order that any effect of age class could be considered for each individual in isolation, without potentially confounding effects from a partner. Females can raise offspring without the assistance of a male (Scott 1998). Ten beetles were allocated to each age class and paired at the appropriate time for breeding. Beetles were mated (males were aged 2 weeks for all experimental groups) and the male then removed prior to presenting a mouse carcass in order that results were not confounded by his presence (Cotter and Kilner 2010a). On day 4 of the breeding bout (bout duration is from carcass presentation to larval dispersal), hemolymph samples were collected and processed to determine PO levels. Hemolymph samples were obtained from 51 individuals. Day 4 of the breeding bout is a time of intense larval care and lytic activity peaks at this time (Cotter et al. 2013).

#### Changes in personal immunity (AMP, defensin) across lifespan in breeding and non‐breeding beetles

Potential changes in defensin expression across lifespan provide us with a proxy for investment into the humoral arm of the personal immune system as the organism ages. Due to the nature of humoral immunity and the fact that it is largely induced upon challenge (defensin expression is low or absent in unchallenged individuals), in this part of the study, all individuals were challenged with an immune elicitor in order to upregulate defensin expression. Female beetles were assigned to three age classes: 2, 5, and 8 weeks. Within each age class, beetles were split into either breeding or nonbreeding subgroups. This resulted in six groups, with nine individuals per group. The immune elicitor comprised 1mg of lipopolysaccharide (LPS) (Sigma‐Aldrich, Dorset, UK) and 2.5 mg of peptidoglycan (PEP) (Sigma‐Aldrich, Dorset, UK) suspended in 1ml of sterile insect ringer's solution. 1 ul of this solution was injected into the cuticle behind the pronotum using a Hamilton syringe. Injections occurred 24 hours prior to haemolymph sampling (to upregulate defensin to the greatest extent (Reavey et al. 2014)) and in the case of the breeding beetles this occurred on day 3 of the breeding bout, with sampling taking place on day 4. Males for all experimental groups were 2 weeks old and were removed after mating. RNA was extracted from the beetles, and defensin upregulation was measured in accordance with the protocol below. Total body tissue from each beetle was pooled during extraction (to maximize samples with a given extraction effort); three beetles were pooled resulting in three overall samples per group. Due to death in the week eight group, the sample size was diminished (six beetles of the initial nine survived in both the breeding and nonbreeding group). One sample was omitted from the 2‐week‐old breeding beetle experimental group due to potential error introduced during the extraction process.

#### Changes in social immunity across lifespan in breeding beetles

Lytic activity was measured in breeding beetles across lifespan. Lytic activity is only upregulated in the presence of a breeding resource (Cotter and Kilner 2010a). Six age classes were used, beetles aged 2–7 weeks at weekly intervals, with each age class consisting of a discrete group of female beetles. Ten beetles were allocated to each age class and paired at the appropriate time for breeding (males were 2 weeks old for all experimental groups). Beetles were mated and the male then removed prior to presenting a mouse carcass in order that results were not confounded by his presence (Cotter and Kilner 2010a). On day 4 of the breeding bout, exudate samples were obtained from all beetles and processed to determine lytic activity levels. Exudate samples were obtained from 51 individuals.

### Experiment 2: The effect of wounding on immunosenescence

In conjunction with measuring PO and lytic activity in breeding beetles across lifespan (Experiment 1), a manipulative experiment was also carried out to determine whether wounding with a sterile 0.5‐mm needle at various stages of lifespan affected the trade‐off between personal and social immunity (Cotter et al. 2013). The experimental setup was as described in Experiment 1c, except that a further group of beetles for each age class was used to test the effects of wounding. On day 3 of the breeding bout, the beetles in the wounded treatment group were wounded on the cuticle behind the pronotum with a sterile 0.5‐mm needle, while those in the non‐wounded group were handled. On day 4 of the breeding bout, exudate samples and hemolymph samples were obtained from all beetles and processed to determine lytic activity and PO levels. Exudate samples and hemolymph samples were obtained from 94 individuals, predominantly due to mortality in the later groups. This enabled us to consider whether immune insult through wounding at different age classes (shown previously to upregulate PO (Reavey et al. 2014) while downregulating lytic activity (Cotter et al. 2013)) results in a change in the balance of personal and social immunity across lifespan.

### Hemolymph sampling

Hemolymph was obtained from *N. vespilloides* by piercing the cuticle behind the pronotum with a sterile 0.5‐mm needle and then collecting the hemolymph as it was released with a pipette (approximately 5 μL hemolymph is released). The hemolymph was then diluted with an equal volume of anticoagulant buffer to prevent it from forming a solid mass (EDTA anticoagulant in PBS – pH 7.4) and then stored in a freezer (−20°C) prior to analysis.

### Phenoloxidase (PO) assay

Following defrosting of the hemolymph samples, 2 μL of hemolymph/anticoagulant buffer solution was added to 500 μL of PBS (pH 7.4). 100 μL of this solution was placed in a well of a 96‐well microplate with 100 μL of 10 mmol/L dopamine. While many researchers use L‐dopa as a substrate for PO reactions, for insect POs, dopamine is the preferred substrate over L‐dopa. It is the natural substrate for insects and is more soluble than L‐dopa (Sugumaran 1998). Readings were taken every 10 sec for 3 min at 490 nm and 25°C on a Thermo Scientific Multiscan Spectrum spectrophotometer. The maximum rate of reaction across six windows of change (absorbance readings) was then used as an approximation of PO level.

### Exudate sampling

When disturbed or handled, most of the beetles produce an exudate from their abdomen. Tapping the abdomen gently often results in the production of exudate. This can then be collected in a capillary tube, blown into an Eppendorf, and stored until processing. Lytic activity of the samples was measured as described below.

### Lytic assay

Bacterial agar plates were used and clear zones measured to determine lytic activity. The agar plates consisted of 10 mL of 1.5% agar: potassium phosphate buffer (2:1) and 50 mg of freeze‐dried *Micrococcus luteus*. *Micrococcus luteus* was selected as it is a soil bacterium, which is the breeding environment of the burying beetle. The exudate samples were processed by punching twenty 2‐mm‐diameter holes in each agar plate and filling each well with 1 μL of exudate. Two technical replicates were processed per sample. The plates were incubated at 33°C for 24 h, and the resulting clear zones were measured using digital callipers to determine the magnitude of lytic activity. Lytic activity (mg/mL) was then calculated from a standard serial dilution of hen egg white lysozyme.

### Antimicrobial peptide (AMP) assay

Due to its ubiquity, we chose the AMP *defensin* as our measure of humoral immunity. RNA was extracted 24 h after injection of the elicitor and qRT‐PCR used to determine any changes in *defensin* expression across the age classes and with breeding status. RNA was isolated using Trizol^®^ Reagent (Invitrogen, ThermoFisher Scientific) in accordance with the manufacturer's instructions. DNA was removed by treatment with TURBO^™^ DNase (Invitrogen, ThermoFisher Scientific) and RNA converted to cDNA using a High Capacity RNA‐to‐cDNA kit (Applied Biosystems, ThermoFisher Scientific). Primers were designed for *defensin* and the housekeeping gene *beta‐tubulin* from ESTs (expressed sequence tags) known for *N. vespilloides* (Vogel et al. 2011) (See Supplementary information). 10 μL of SYBR, 0.4 μL FWD primer, 0.4 μL REV primer, 7.2 μL of water, and 2 μL of 25 ng/μL of cDNA were used in each PCR. Real‐time PCR was carried out using a Biorad Thermo Cycler with the following conditions: 95°C for 3 min, and 50× (95°C for 10 sec, 52°C for 10 sec, and 72°C for 20 sec) with a melt analysis from 65 to 95°C ramping at 0.5°C. Primer efficiency (PCR efficiency as other conditions were constant) was determined using a feature on the thermo cycler machine, for use in the data analysis (*defensin*: 1.9, *tubulin*: 2.0). The Pfaffl equation was used as the model for data analysis.

### Statistical analyses

All statistical analyses were carried out in R 3.1.3 (R Development Core Team, 2014). General linear mixed models were used in all analyses to control for the effect of family, apart from Experiment 1b where a generalized least squares model was carried out due to the unequal variance. In Experiment 1b, values from the Pfaffl equation were normalized for use in the model. The assumptions of the models were tested by visual inspection of the diagnostic plots. PO and lytic activity data were log‐transformed to approximate normality. The statistics presented are estimations from the minimum adequate model following stepwise deletion of nonsignificant variables, that is, the model only contains variables that are significant, unless statistics for nonsignificant terms are quoted, in which case the nonsignificant term is included last in the model.

## Results

### Experiment 1: Changes in personal and social immunity across lifespan

#### Changes in personal immunity (PO) across lifespan in breeding and non‐breeding beetles

##### Non‐breeders

**Figure 2:**
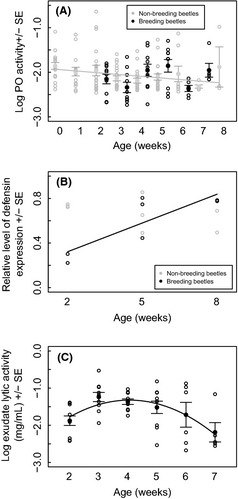
Changes in personal and social immunity across lifespan, (A) the relationship between PO activity and age in non‐breeding and breeding beetles (Experiment 1a). The raw data for PO are in open gray circles for the non‐breeding beetles and open black circles for breeding beetles against the age in weeks of the beetle. Means and SEs are shown for the raw data, alongside a fitted line of the model in gray for the relationship between age and PO activity in non‐breeders. (B) The relative level of defensin expression against the age in weeks of the beetles (Experiment 1b). Raw data for non‐breeding female beetles are shown in gray circles and for breeding female beetles in black circles. The fitted line of the model for defensin expression in breeders with age is included in black. (C) Lytic activity against beetle age (Experiment 1c). Raw data are presented in open black circles. The data are produced from female beetles. The line shows the fitted values of the model across lifespan.

PO levels decreased across lifespan in non‐breeding beetles in a linear manner, dropping as the beetle aged (GLMM: estimate = −0.035 ± 0.015, *t*_119_ = −2.31, *P* = 0.023; Fig. 2A). There was no effect of sex on PO levels (GLMM: estimate = 0.093 ± 0.065, *t*_126_ = 1.43, *P* = 0.155) or the age × sex interaction (GLMM: estimate = 0.025 ± 0.031, *t*_125_ = 0.80, *P* = 0.425). Beetles were also analyzed from 0 to 4 weeks in order that selection for long‐lived beetles was not occurring (age may correlate with PO), and due to small sample sizes for the later groups. PO levels still decreased across the lifespan of a beetle (GLMM: estimate = −0.081 ± 0.027, *t*_82_ = −3.06, *P* = 0.003). No effect of sex on PO was observed (GLMM: estimate = 0.048 ± 0.075, *t*_86_ = 0.64, *P* = 0.526) or the age × sex interaction (GLMM: estimate = −0.057 ± 0.054, *t*_85_ = −1.06, *P* = 0.291).

##### Breeders

In contrast, age did not affect PO levels in breeding beetles (GLMM: estimate = 0.048 ± 0.035, *t*_47.89_ = 1.37, *P* = 0.176; Fig. 2A).

#### Changes in personal immunity (AMP, defensin) across lifespan in breeding and non‐breeding beetles

Defensin levels increased with age for breeding beetles, but there was no change in expression with age for nonbreeding beetles, as observed in the age × breeding status interaction (*F*_1,11_ = 13.13, *P* = 0.004, Fig. 2B).

#### Changes in social immunity across lifespan in breeding beetles

Lytic activity initially increased until female beetles were around 4 weeks of age, before decreasing as the beetles aged further (GLMM: age = 0.864 ± 0.243, *t*_44.13_ = 3.55, *P* < 0.001, age^2^ = −0.106 ± 0.028, *t*_44.87_ = −3.85, *P* < 0.001; Fig. 2C).

### Experiment 2: The effect of wounding on immunosenescence

**Figure 3:**
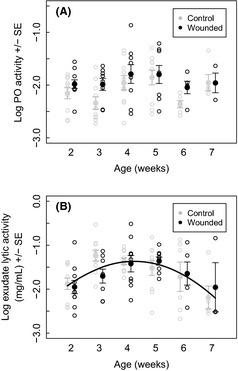
The effect of wounding on immunosenescence, (A) PO activity and (B) Lytic activity against beetle age (weeks). The line shows the fitted values of the model across lifespan. For both plots controls are shown in gray and wounded beetles in black. The solid circles and error bars represent the means and SEs of the raw data, while the raw data are presented in the respective colors in open circles. The data are produced from female beetles.

PO was upregulated following wounding (GLMM: estimate = 0.189 ± 0.082, *t*_85.84_ = 2.293, *P* = 0.024; Fig. 3A), and this effect did not change significantly with age (age × wounded interaction: GLMM: estimate = −0.029 ± 0.052, *t*_84.26_ = −0.570, *P* = 0.570; Fig. 3A). There was no effect of wounding on lytic activity (GLMM: estimate = −0.061 ± 0.101, *t*_90_ = −0.609, *P* = 0.544; Fig. 3B) and no interaction between age and wounding (GLMM: estimate = −0.053 ± 0.370, *t*_88_ = −0.144, *P* = 0.886), or age^2^ and wounding (GLMM: estimate = 0.017 ± 0.043, *t*_88_ = 0.402, *P* = 0.688). There is no correlation between PO and lysozyme activity for either wounded (*F*_1,41_ = 2.55, *P* = 0.118) or non‐wounded beetles (*F*_1,47_ = 1.24, *P* = 0.272).

## Discussion

Here, to the best of our knowledge, for the first time in any taxa, we assess immunosenescence in both personal and social immunity. We show that while personal immunity is maintained (defensin) or declines (PO) with age in non‐breeders, breeding beetles maintain (PO) or even increase (defensin) their investment in personal immunity. Social immunity on the other hand, which is present only in breeding beetles, peaks in middle‐aged beetles before starting to fall as beetles age.

As hypothesized, PO was found to decline with lifespan in non‐breeding *N. vespilloides*, the pattern occurring in both sexes. This species seems to follow a “typical” pattern of immunosenescence, the decline of immune function as the organism ages. Indeed, it supports other studies across taxa showing a decline in PO across lifespan (Adamo et al. 2001; Moret and Schmid‐Hempel 2009; Whitehorn et al. 2011; Roberts and Hughes 2014). However, in our experiment, PO decreases even in very young beetles, with the highest activity occurring just after emergence and declining steadily throughout life. As these beetles have a pre‐reproductive period of approximately 2 weeks, the highest levels of investment correlate with the period before they have the opportunity to reproduce. From an investment perspective, a younger organism is selected to invest in their immune system to aid chances of survival to adulthood and future breeding opportunities. The initially high PO levels could also be due to sclerotization of the cuticle, which is relatively soft immediately after eclosion. Also, due to the soft cuticle forming a less effective barrier to microbes, younger beetles may be selected to invest more in immune function. However, this would only be relevant for the first 2 days post‐emergence as the cuticle hardens rapidly after eclosion. In older organisms, due to a limited duration of lifespan ahead, the optimal strategy may be to conserve resources for reproduction. Furthermore, older individuals may be constrained further by a decline in condition and damage to tissue with age.

The finding that PO does not decline in female breeding beetles was initially surprising; PO declines in nonbreeders and is suppressed in young breeding beetles (Reavey et al. 2014). As the experiments on non‐breeding and breeding beetles were carried out at different times, an element of caution must be used when comparing results. However, it appears that PO is indeed downregulated in young breeding beetles (2–3 weeks) relative to PO in virgins of the same age but then maintained or upregulated in older breeding beetles. The fact that PO is held at the same level during breeding across lifespan or indeed *upregulated* compared to the decline in non‐breeders indicates that personal immunity may be important when beetles gain their first breeding opportunity in later life. Although PO is suppressed during breeding in young beetles, perhaps a further decline in older beetles would fully compromise standing immunity at a time when the individual is investing heavily in lifetime reproductive success. However, it may be that a breeding attempt, which could be the final opportunity to reproduce, calls for the organism to invest both in the brood, but also in “staying alive” for the duration of the parental care period.

Considering our measure of humoral immunity, defensin expression following an immune challenge was found to remain at constant expression levels throughout lifespan in female non‐breeders. A limited sample size and only scope for three age classes mean that we must be cautious when interpreting the results. However, although the variation is high, we know that gene expression studies using a range of methodologies across taxa show that some of the most dramatic transcriptional changes that occur during aging are associated with immunity, and so this variation may be expected (DeVeale et al. 2004). It seems that age, encompassing a decline in state, does not affect humoral investment as measured by defensin expression in non‐breeders. Knowledge on how PO investment and defensin investment compare with regard to costs and benefits would be interesting, considering that PO declines with age (albeit PO in unchallenged individuals). However, levels of defensin expression increase in breeding beetles across lifespan. An increase in immune response genes with age has also been observed in *Drosophila* (Pletcher et al. 2002; Zerofsky et al. 2005). Also, the process of mating has been shown to increase AMP expression (Peng et al. 2005). If this is occurring in *N. vespilloides*, perhaps mating is differentially affecting female immunity in different age classes. It is of note that the levels of defensin expression for breeding beetles at younger age classes are lower that than of non‐breeders. PO is suppressed during breeding (Reavey et al. 2014), and this may also be occurring for humoral immunity, with the suppression lifted at older age classes when all resources may be invested in both reproduction and “staying alive” to complete the breeding bout.

Lytic activity, the social immune response (Cotter and Kilner 2010b), increased in female breeding burying beetles up to middle age, before decreasing in old age. Different patterns of reproductive investment with age exist across taxa, with a common pattern being an initial increase in investment in early–middle‐aged class, before a decline in old age. Hypotheses for these changes include the following: the selection hypothesis (Curio 1983; Mauck et al. 2004), the constraint hypothesis (Curio 1983; Kornduer 1996; Pärt 2001), and the restraint hypothesis (Williams 1966; McNamara et al. 2009). As there was no significant mortality in our experimental beetles up to 5 weeks, which would allow less‐fit individuals to be removed, the selection hypothesis does not support the initial increase in lytic activity with age. It is more likely that the constraint or restraint hypotheses support the changes we observe in lytic activity. For example, there may be physiological constraints present with regard to lysozyme production; this process may require maturation, and indeed, the age at which the beetles normally produce their first brood in the field is unknown. The restraint hypothesis provides another possible explanation for changes in reproductive investment: Young individuals provide less reproductive effort as the value of the first/early brood is lower than that of expected future offspring. Life‐history theory predicts increased reproductive effort when residual reproductive value decreases (Trivers 1972). There is evidence of reproductive restraint in burying beetles (Cotter et al. 2010a), and elements of this theory may apply to the changes in lytic activity: For young individuals (2 weeks), the value of the brood is low relative to future broods and may not merit such a high investment in lysozyme production, which is a costly resource (Cotter et al. 2010b). While the pattern of lytic activity differs slightly to that of first‐time reproductive investment in this species (Cotter et al. 2010a), the common pattern is for breeding performance to improve in the early years of life, reaching a maximum at middle age (Reid et al. 2003), which is exactly the pattern observed for lytic activity.

With regard to the effect of immune challenge on personal and social immunity, we see that wounding upregulates PO in female breeding beetles across lifespan. This supports data, showing beetles can still upregulate PO while breeding (Reavey et al. 2014). The fact that the response is of a similar magnitude as the organism ages may indicate the importance of responding to a challenge at any age while breeding. In contrast, when considering changes in lytic activity in response to wounding across lifespan in female beetles, no effect of wounding was observed. We initially thought this was odd as the experiment was based on the results of Cotter et al. (2013) with the expectation that personal and social immunity would trade‐off. However, on closer inspection (Fig. 3B), it can be observed in our study that this trade‐off exists only in week 3 beetles, which is the age class used in the Cotter et al. (2013) experiment. Why this trade‐off exists at this age and none of the other age classes is unclear; it may be that at other ages a trade‐off occurs with different traits or that as lytic activity is lower at other age classes, it is not as costly and does not require a decrease in response to wounding.

Future experiments should consider whether the response of PO to wounding in non‐breeders changes with age. This was not considered as the focus of this study was initially on whether the trade‐off between personal and social immunity (only present in breeding beetles) changed with age. Furthermore, measurements of both proPO and PO could be of interest as they might show different patterns with age (e.g., Armitage and Boomsma 2010). Changes in lytic activity in male burying beetles with age would also be interesting. Males have lower lytic activity levels than females (Cotter and Kilner 2010a); we might expect a similar pattern, but lower absolute levels. The responses to different immune challenges would be interesting to observe. It would also be useful to measure a greater number of AMPs.

In summary, both personal and social immunity change across lifespan, but how they change depends upon the immune traits measured and the breeding status of the individual. These changes are likely a result of the decline of the organism alongside strategic changes in immune investment with age. While senescence is not an adaptive process, and indeed in the wild animals generally do not live long enough for senescence to be the cause of mortality, some patterns of decline may be adaptive responses due to “time left to further fitness,” resulting in changes in resource allocation and immune trait expression.

Our results regarding PO in non‐breeders generally support other findings in the literature, suggesting that the decline with age may be a conserved strategy across species. Changes in PO with age in taxa while breeding has not been researched in detail; the long breeding bout in burying beetles lends itself to its examination. The maintenance/upregulation of defensin is also similar to immune response gene studies in the literature, where it seems the transcripts often are at high levels in older age classes. To the best of our knowledge, our study is the first to consider changes in social immunity with age in a reproductive insect. As study on the area of social immunity is fairly recent, as research in this field grows, further studies across taxa will yield interesting findings with regard to how much variability in the pattern exists and what drives the trends. Age‐related investment in immune function contributes to how well an organism can resist or moderate infection at various stages of their lifespan, which has consequences for host parasite dynamics. Recognizing changes in immune function, both personal and social, with age is important both for understanding evolutionary theory and providing clues regarding factors affecting animal health.

## Conflict of Interest

None declared.

## Supporting information


**Data S1.** Primer design.
