# Supplementary material for: Aging in personal and social immunity: do immune traits senesce at the same rate?
Source: Ecol Evol. 2015 Sep 18;5(19):4365–75. doi: 10.1002/ece3.1668 (PMC4667822; doi:10.1002/ece3.1668)
Supplement: Supplementary file 1 — Data S1. Primer design. [file ECE3-5-4365-s001.docx]

## Supplementary information

### Primer design

***Nicrophorus vespilloides* Tubulin Accession Number (HO113191.1)**

ATCCAGAGCAGTTGATAACCGGAAAGGAAGATGCAGCCAACAATTACGCTCGTGGTCATTACACCATCGGGAAAGAGCTCATCGATCAGGTATCTGATAGGATTCGCAAACTGGCGGATCAATGTCAAGGACTTCAGGGTTTCCTGATTTTCCACTCTTTTGGAGGAGGGACCGGTTCCGGTTTTACTTCCCTTTTGATGGAAAGGTTGCCAGTGGATTACGGAAAGAAGAGCAAACTGGAATTTGCCGTTTACCCGGCGCCTCAGGTCTCAACTGCTGTGGTGGAGCCCTACAATTCGATCCTTACCACGCACACCACCCTTGAACACTCCGATTGCGCCTTTATGGTAGACAATGAAGCAATCTACGATATCTGCTTGAAGAATTTGGATATCCCTAGACCAGGATACTTGAATCTCAACAGACTCATCAGTCAGATCGTTTCATCTACGACCGCATCTTTGAGATTCGATGGAGCCATGAACGTCCATCTTACGGAATTCCAAACGAATTTAGTTCCTTACCCACGTATACATTTTCCATTAATGACTTATGCACCAATCATTTCAGCAGCGAAAGCCTACCACGAACAAATCTCAGTAGCCGAAATCACAAACGCGTGCTTCGAACCCAACAACCAGATGGTGAAATGTGATCCTCGTCGAGGAAAG

Order

>Tub183Fwd: GCGGATCAATGTCAAGGACT (Tm = 57)

>Tub183Rev: ATTGTAGGGCTCCACCACAG (Tm = 57)

**Defensin (NIC-SSH_ContigX1) Accession Number ()**

GATGGTTGCCAGCTTCGTGAGCGCTGGACCGGTTGAGCAAGATGCCGAAGGACATGTTGTGGAAAGGGCCAACAGGCAACGCAGGGTGACCTGCGATTTATTGAGCGTATCGACGCCCTACGGTTCCGTCAACCATTCGGTCTGCGCCGCCCACTGCCTCGCCATGCTGAAGGGTTTCAGAGGTGGAAGATGCATCGACGGAGTCTGCAATTGCAGGAAGTAAAGGTGTTGTCGATTAATTGACTTCCACCGATTGGACAATTGCCTCGATTGGAAGAGACCCCCTAAACAGCTTTAATCCCACAAGTTAATTAAATTAGGTAACGAAAAAAAAGAAGTTTGCAATAAATAAAACGTAGTTGTTACAAAAAAAAAAAAAA

Order

>Def199Fwd: CAGGGTGACCTGCGATTTAT (Tm = 57)

>Def199Rev: TCTCTTCCAATCGAGGCAAT (Tm = 57)
